# Complete genome sequence of shrimp hemocyte iridescent virus (SHIV) isolated from white leg shrimp, *Litopenaeus vannamei*

**DOI:** 10.1007/s00705-017-3642-4

**Published:** 2017-11-27

**Authors:** Liang Qiu, Meng-Meng Chen, Ruo-Yu Wang, Xiao-Yuan Wan, Chen Li, Qing-Li Zhang, Xuan Dong, Bing Yang, Jian-Hai Xiang, Jie Huang

**Affiliations:** 1 Qingdao Key Laboratory of Mariculture Epidemiology and Biosecurity, Key Laboratory of Maricultural Organism Disease Control, Ministry of Agriculture, Function Laboratory for Marine Fisheries Science and Food Production Processes, Qingdao National Laboratory for Marine Science and Technology, Yellow Sea Fisheries Research Institute, Chinese Academy of Fishery Sciences, Qingdao, 266071 China; 2 Shanghai Ocean University, Shanghai, 201306 China; 3 Dalian Ocean University, Dalian, 116023 China; 4 Key Laboratory of Experimental Marine Biology, Institute of Oceanology, Chinese Academy of Sciences, Qingdao, 266071 China

## Abstract

Infection with shrimp hemocyte iridescent virus (SHIV), a new virus of the family *Iridoviridae* isolated in China, results in a high mortality rate in white leg shrimp (*Litopenaeus vannamei*). The complete genome sequence of SHIV was determined and analyzed in this study. The genomic DNA was 165,809 bp long with 34.6% G+C content and 170 open reading frames (ORFs). Dotplot analysis showed that the longest repetitive region was 320 bp in length, including 11 repetitions of an 18-bp sequence and 3.1 repetitions of a 39-bp sequence. Two phylogenetic trees were constructed based on 27 or 16 concatenated sequences of proteins encoded by genes that are conserved between SHIV homologous and other iridescent viruses. The results of this study, suggest that SHIV should be considered a member of the proposed new genus “*Xiairidovirus”.*

**Electronic supplementary material:**

The online version of this article (10.1007/s00705-017-3642-4) contains supplementary material, which is available to authorized users.

The *Iridoviridae* are a family of large, icosahedral viruses with double-stranded DNA genomes ranging in size from 103 to 220 kbp. The iridescent viruses of the subfamily *Alphairidovirinae* infect ectothermic vertebrates (bony fish, amphibians, and reptiles), whereas members of the subfamily *Betairidovirinae* mainly infect insects and crustaceans [1]. The iridescent viruses contain circularly permutated and terminally redundant double-stranded genomes [2, 3]. To date, the complete genomes of 39 iridescent viruses have been sequenced (Supplemental File 1). Twenty of these are members of the genus *Ranavirus*, seven are members of the genus *Megalocytivirus*, three are members of the genus *Lymphocystivirus*, seven are members of the genus *Iridovirus*, and one is a member of the genus *Chloriridovirus.*

Shrimp hemocyte iridescent virus (SHIV) caused severe disease and high mortality in farmed *Litopenaeus vannamei* in December of 2014 in Zhejiang Province in China and was isolated and identified by Qiu et al. in 2017 [4]. PCR test results showed that farmed *L. vannamei*, *Fenneropenaeus chinensis*, and *Macrobrachium rosenbergii* were SHIV positive, and the detection rate was 15.8% in farmed shrimp samples collected from 2014 to 2016, indicating that SHIV is a new threat to the shrimp culture industry in China. SHIV exhibited a typical icosahedral structure with a mean diameter of 158.6 ± 12.5 nm (n = 30). Phylogenetic analysis using amino acid sequences of the major capsid protein (MCP) and ATPase indicated that it belongs to the proposed genus “*Xiairidovirus”.* [4]. Since only the MCP and ATPase sequences of SHIV were determined previously (accession numbers KY681039 and KY681040), the relationship of the complete genome sequence SHIV to those of other iridescent viruses has not yet been examined.

The nucleotide sequence of the SHIV genome was determined by the viral metagenomics sequencing method described by Qiu et al. [4], using an Illumina HiSeq 2500 (PE125) instrument. Gaps between assembled fragments were filled and the complete genome sequence was verified using the primer walking method (Fig. 1a). Information about the primers used in this study is shown in Supplemental File 2. DNA sequencing reads corresponding to SHIV sequences were mapped across the length of the genome (Fig. 1b). The sequencing coverage was 100%, and the fold coverage per base ranged from 101 to 118,328, with an average of 6364.7, indicating that the sequencing data completely covered the SHIV genome. The sequencing depth was uneven in distribution, possibly because of preferences in sequencing and amplification. The complete genome sequence of SHIV obtained from the original tissue has been deposited in the GenBank database (accession number MF599468).

**Fig. 1:**
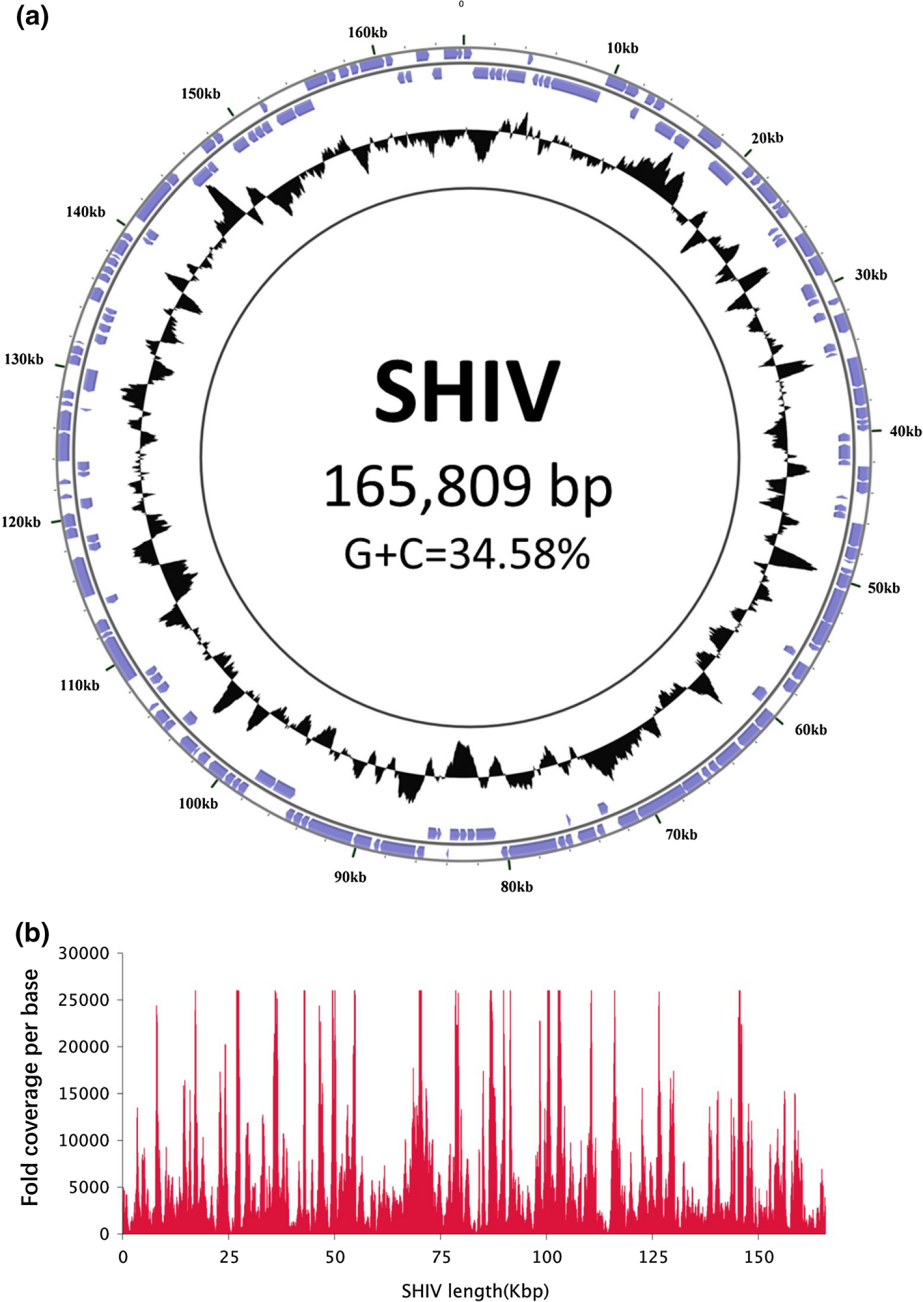
**a.** Circular map of the 165,809-bp SHIV genome. The outer scale is numbered clockwise in bp. Circles 1 and 2 (from outside to inside) show ORFs on the forward and reverse strand, respectively). Circle 3 represents the G+C content along the genome sequence. Peaks toward the outside indicate that the G+C content is greater than 50%; peaks toward the inside indicate that the G+C content is lower than 50%; the height is proportional to the content). **b.** Sequence coverage of SHIV genomic sequences. Illumina reads were mapped to the assembled SHIV sequence using the software BWA 0.7.8. Sequence coverage was calculated according to the number of times each base mapped to the SHIV genome

The SHIV genome consists of a double-stranded DNA molecule of 165,809 bp. A comparison with other members of the family *Iridoviridae* showed that the SHIV genome is larger than those of members of the genera *Ranavirus* (103,681-140,131 bp) and *Megalocytivirus* (110,104-112,636 bp) but smaller than those of members of the genera *Iridovirus* (197,693-220,222 bp) and the chloriridovirus IIV-3 (191,100 bp). Comparison with the three sequenced members of the genus *Lymphocystivirus* showed that the SHIV genome is much larger than that of LCDV-1 (102,653 bp) but smaller than those of LCDV-Sa (208,501 bp) and LCDV-C (186,250 bp) (Supplemental File 1). The G+C ratio of the SHIV genome is 34.6%, which is higher than that of the members of the genera *Iridovirus* (28.1-30.9%), except for IIV-31 (35.09%), and *Lymphocystis* (27.3-33.0%) but lower than that of the members of the genera *Megalocytivirus* (53.0-55.0%) and *Ranavirus* (48.6-57.0%) and the chloriridovirus IIV-3 (47.89%) (Supplemental File 1).

To date, five iridescent viruses were found in crustaceans [4]. However, IIV-31, which was isolated from the pill bug, *Armadillidium vulgare* [5, 6], is the only one for which a complete genome sequence is available. IIV-31 has the largest genome size (220,222 bp) among all sequenced viruses of the family *Iridoviridae* and is much larger than SHIV, but the G+C content of IIV-31 (35.1%) is relatively close to that of SHIV.

We did a dotplot analysis of the SHIV genome using Genious 10.2.2. Like other iridescent viruses, the SHIV genome was found to contain numerous repetitive sequences. The longest repetitive region was identified at nt position 27,052 to 27,371 (ORF 29R). The 320-bp repetitive sequence consists of 11 repetitions of CAACCACCACAGAACCTC and 3.1 repetitions of CAACCGAATCCATTGAAATGGAAAATGTAACATCCACCA. There were some direct, inverted and palindromic repetitive sequences located at positions 113,519 to 113,693 (non-coding region) and 114,420 to 114,564 (non-coding region). The biological function of these repetitive sequences remains to be determined.

One hundred seventy open reading frames (ORFs) encoding proteins ranging from 21 to 1,167 amino acids were identified in the SHIV genome using the software GeneMark (http://topaz.gatech.edu/GeneMark/). Of the 170 ORFs, 102 were in the forward orientation (R), and 68 were in the reverse orientation (L) (Fig. 1a and Supplement File 3). The number of ORFs predicted for SHIV (170) is fewer than that of most members of the genus *Iridovirus* (167-468) and all three sequenced members of the genus *Lymphocystivirus* (183-240) but more than that of members of the genera *Ranavirus* (91-162), *Megalocytivirus* (108-135), and *Chloriridovirus* (126) (Supplemental File 1). There are 11 pairs of overlapping ORFs in the SHIV genome: 3L/4L, 6L/7R, 24R/25R, 34L/35R, 41R/42R, 56R/57R, 68R/69R, 84L/85L, 93R/94L, 148L/149L, and 169R/170R. Nine of these 11 pairs of ORFs have an overlap of 1-7 bp, which is similar to what has been reported for TRBIV, ISKNV, OSGIV, ATV, and SGIV [7].

Analysis using Blast2GO to identify putative proteins revealed that 63 ORFs (37.1%) had been annotated with known functions, including enzymes and structural proteins including the ATPase, DNA polymerase and major capsid protein. A BLASTp analysis using sequences from members of the family *Iridoviridae* showed that 22 ORFs were most similar to genes of IIV-31, the only iridescent virus found in crustaceans for which a genome sequence is available. Nineteen ORFs were most similar to the genes of IIV-6 and LCDV-Sa, which were isolated from the stem-boring lepidopterans *Chilo suppressalis* [8] and *Sparus aurata* [9], respectively. The results show that SHIV might be comparatively closely related to IIV-31, IIV-6, and LCDV-Sa.

Of the 170 ORFs in the SHIV genome, 27 (Supplemental File 3) showed sequence similarity to regions of 15 viral genomes representing all five genera of the family *Iridoviridae*, suggesting that these 27 ORFs are conserved and can be used for phylogenetic analysis of SHIV with other iridescent viruses (Supplemental File 4). In a previous report, Eaton et al. [10] reported 26 conserved genes that are present in every iridescent virus sequenced up to the time of the report. Like the 26 genes reported by Eaton et al. [10], the majority of the 27 conserved genes identified in this study (24 of 27 ORFs) have a predicted function based on sequence similarity of their encoded proteins to other characterized proteins. Some of these genes, including those encoding the membrane protein, RNA polymerase, NTPase, RAD2, ICP-46, MCP, deoxynucleoside kinase, ribonuclease III, DNA polymerase, phosphotransferase, and tyrosine kinase, are present among the 26 genes reported previously as well as the 27 genes identified in this study.

Twenty-seven conserved SHIV ORFs and the orthologous genes from 15 completely sequenced iridescent viruses of five genera were used to generate a phylogenetic tree (Fig. 2a). We further selected 16 of the 27 conserved genes and the orthologous genes from 34 completely sequenced iridescent viruses to establish a more complete phylogenetic tree of the family *Iridoviridae* (Fig. 2b). Both phylogenetic trees showed that the members of the five genera of the family *Iridoviridae* formed separate branches, and there was high bootstrap support (100%) for SHIV forming a new branch in the subfamily *Betairidovirinae*. Qiu et al. [4] suggested assigning SHIV to a new genus, tentatively named “*Xiairidovirus*” that includes iridescent viruses that infect shrimp, lobster or crayfish. The findings in this study support this proposal.

**Fig. 2:**
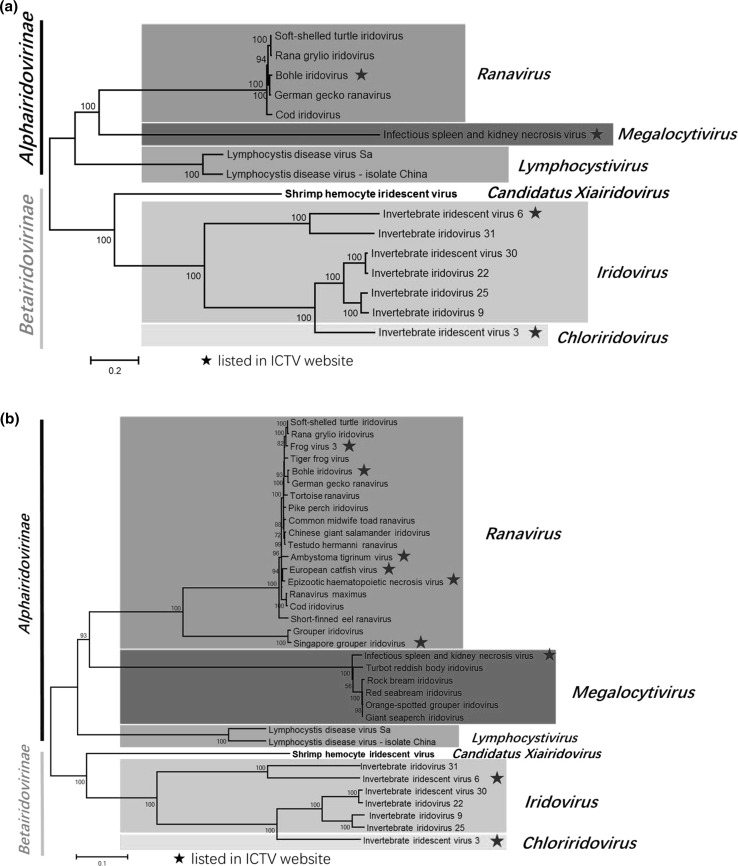
Concatenated phylogenetic tree of iridescent viruses. **a.** Twenty-seven conserved genes from 15 other completely sequenced iridescent viruses were rearranged as continuous amino acid sequences with the same order as in SHIV. A multiple sequence alignment was carried out using MUSCLE. The tree was reconstructed by the maximum-likelihood method using MEGA 5.0 and the numbers indicate percentage bootstrap support. **b.** Sixteen of the 27 conserved genes from 34 completely sequenced iridescent viruses underwent the same process to establish a tree that includes most of the completely sequenced viruses of the family *Iridoviridae*. Percentage bootstrap values (1000 replicates) are shown. Bar, expected nucleotide substitutions per site

In the two phylogenetic trees, four members of the genus *Iridovirus* (IIV-9, IIV-22, IIV-25, and IIV-30) and one member of the genus *Chloriridovirus* (IIV-3) were in the same branch. Similarly, phylogenetic analysis of proteins encoded by IIV3, IIV6, and IIV9 revealed that IIV9 is more closely related to IIV3 than to IIV6 [11–13]. This was also true of IIV22, IIV22A, IIV25, and IIV30, four close relatives of IIV9 [14–17], indicating that some members of the genus *Iridovirus* are more closely related to members of the genus *Chloriridovirus* than to other iridoviruses from insects [18]. Only two species in the genus *Iridovirus*, *Invertebrate iridescent virus 1* and *Invertebrate iridescent virus 6*, have been recognized by the International Committee on Taxonomy of Viruses (ICTV). More biological and genomic data are needed before it can be determined whether other related viruses belong to the genus *Iridovirus* [18]. Based on the phylogenetic analysis results available to date, we suggested that IIV-9, IIV-22, IIV-25, and IIV-30 be assigned to the genus *Chloriridovirus*. Our study provides new information for future studies of the epidemiology of SHIV and the taxonomy and evolution of the family *Iridoviridae*.

## Electronic supplementary material

Below is the link to the electronic supplementary material.

Supplementary material 1 (TXT 161 kb)

Supplementary material 2 (DOCX 45 kb)

## References

[1] Gregory CV, Hick P, Ince IA, Jancovich JK, Marschang R, Qin Q, Subramaniam K, Waltzek TB, Whittington R, Williams T, Zhang QY, Ictv Report Consortium (2017). Ictv virus taxonomy profile: iridoviridae. J Gen Virol.

[2] Chinchar VG, Hyatt A, Miyazaki T, Williams T (2009). Family iridoviridae: poor viral relations no longer. Curr Top Microbiol Immunol.

[3] Chinchar VG, Yu KH, Jancovich JK (2011). The molecular biology of frog virus 3 and other iridoviruses infecting cold-blooded vertebrates. Viruses.

[4] Qiu L, Chen MM, Wan XY, Li C, Zhang QL, Wang RY, Cheng DY, Dong X, Yang B, Wang XH, Xiang JH, Huang J (2017). Characterization of a new member of Iridoviridae, Shrimp hemocyte iridescent virus (SHIV), found in white leg shrimp (*Litopenaeus vannamei*). Sci Rep.

[5] Federici B (1980). Isolation of an iridovirus from two terrestrial isopods, the pill bug, *Armadillidium vulgare*, and the sow bug, *Porcellio dilatatus*. J Invertebr Pathol.

[6] Williams T (1994). Comparative studies of iridoviruses: further support for a new classification. Virus Res.

[7] Shi CY, Jia KT, Yang B, Huang J (2010). Complete genome sequence of a Megalocytivirus (family Iridoviridae) associated with turbot mortality in China. Virol J.

[8] Fukaya M, Nasu S (2008). A Chilo iridescent virus (CIV) from the rice stem borer, *Chilo suppressalis* Walker (Lepidoptera: Pyralidae). Appl Entomol Zool.

[9] López-Bueno A, Mavian C, Labella AM, Castro D, Borrego JJ, Alcami A, Alejo A (2016). Concurrence of iridovirus, polyomavirus and a unique member of a new group of fish papillomaviruses in lymphocystis disease affected gilthead seabream. J Virol.

[10] Eaton HE, Metcalf J, Penny E, Tcherepanov V, Upton C, Brunetti CR (2007). Comparative genomic analysis of the family Iridoviridae: re-annotating and defining the core set of iridovirus genes. Virol J.

[11] Wong CK, Young VL, Kleffmann T, Ward VK (2011). Genomic and proteomic analysis of invertebrate iridovirus type 9. J Virol.

[12] Lei XY, Ou T, Zhu RL, Zhang QY (2012). Sequencing and analysis of the complete genome of *Rana grylio* virus (RGV). Arch Virol.

[13] Huang Y, Li S, Zhao Q, Pei G, An X, Guo X, Zhou H, Zhang Z, Zhang J, Tong Y (2015). Isolation and characterization of a novel invertebrate iridovirus from adult *Anopheles minimus* (AMIV) in china. J Invertebr Pathol.

[14] Piégu B, Guizard S, Spears T, Cruaud C, Couloux A, Bideshi DK, Federici BA, Bigot Y (2013). Complete genome sequence of invertebrate iridescent virus 22 isolated from a blackfly larva. J Gen Virol.

[15] Piégu B, Guizard S, Spears T, Cruaud C, Couloux A, Bideshi DK, Federici BA, Bigot Y (2013). Complete genome sequence of invertebrate iridovirus IIV22A, a variant of IIV22, isolated originally from a blackfly larvae. Stand Genom Sci.

[16] Piégu B, Guizard S, Spears T, Cruaud C, Couloux A, Bideshi DK, Federici BA, Bigot Y (2013). Complete genome sequence of invertebrate iridovirus IIV-25 isolated from a blackfly larva. Arch Virol.

[17] Piégu B, Guizard S, Spears T, Cruaud C, Couloux A, Bideshi DK, Federici BA, Bigot Y (2013). Complete genome sequence of invertebrate iridovirus IIV30 isolated from the corn earworm, *Helicoverpa zea*. J Invertebr Pathol.

[18] Piégu B, Guizard S, Spears T, Cruaud C, Couloux A, Bideshi DK, Federici BA, Bigot Y (2014). Genome sequence of a crustacean iridovirus, iiv31, isolated from the pill bug, *Armadillidium vulgare*. J Gen Virol.

