# Supplementary material for: Complete genome sequence of shrimp hemocyte iridescent virus (SHIV) isolated from white leg shrimp, Litopenaeus vannamei
Source: Arch Virol. 2017 Nov 27;163(3):781–5. doi: 10.1007/s00705-017-3642-4 (PMC5814465; doi:10.1007/s00705-017-3642-4)
Supplement: Supplementary file 2 — Supplementary material 2 (DOCX 45 kb) [file 705_2017_3642_MOESM2_ESM.docx]

**Supplemental file 1.** Summary of genomic information of 39 sequenced iridescent viruses. (The virus which contains few homologous genes with SHIV are not listed in the phylogenetic tree and marked in orange)

| **Genus** | **Virus** | **Genome size (bp)** | **G+C content (%)** | **No.**  **of ORFs** | **GenBank Accession no.** |
| --- | --- | --- | --- | --- | --- |
| ***Ca.Xiairidovirus*** | SHIV | 165,809 | 34.6 | 170 | MF599468 |
| ***Ranavirus*** | STIV | 105,809 | 55.1 | 105 | EU627010 |
|  | RGV | 105,791 | 55.1 | 106 | JQ654586 |
|  | FV3 | 105,903 | 55.1 | 98 | AY548484 |
|  | TFV | 105,057 | 55.0 | 105 | AF389451 |
|  | BIV | 103,531 | 55.2 | 100 | KX185156 |
|  | GGRV | 103,681 | 55.1 | 98 | KP266742 |
|  | TRV | 103,876 | 55.2 | 98 | KP266743 |
|  | PPIV | 108,041 | 55.3 | 109 | KX574341 |
|  | CMTV | 106,878 | 55.3 | 100 | JQ231222 |
|  | CGSIV | 105,375 | 55.2 | 111 | KF512820 |
|  | THRV | 105,811 | 55.4 | 98 | KP266741 |
|  | ATV | 106,332 | 54.0 | 91 | NC_005832 |
|  | ECV | 127,751 | 54.2 | 135 | KT989884 |
|  | EHNV | 127,011 | 54.1 | 100 | FJ433873 |
|  | Rmax | 115,510 | 55.2 | 100 | KX574343 |
|  | CoIV | 114,865 | 57.0 | 98 | KX574342 |
|  | SERV | 126,965 | 55.6 | 111 | KX353311 |
|  | GIV | 139,793 | 48.6 | 120 | AY666015 |
|  | SGIV | 140,131 | 48.6 | 162 | AY521625 |
|  | ESV | 127,732 | 54.2 | 136 | JQ724856 |
| ***Megalocytivirus*** | ISKNV | 111,362 | 54.8 | 124 | AF371960 |
|  | TRBIV | 110,104 | 55.0 | 115 | GQ273492 |
|  | RBIV | 112,080 | 53.0 | 118 | AY532606 |
|  | RSIV | 112,590 | 53.0 | 108 | AP017456 |
|  | OSGIV | 112,636 | 54.0 | 121 | AY894343 |
|  | GSIV | 112,565 | 53.0 | 135 | KT804738 |
|  | LYCIV | 111,767 | 53.9 | 126 | AY779031 |
| ***Lymphocystivirus*** | LCDV-Sa | 208,501 | 33.0 | 183 | KX643370 |
|  | LCDV-C | 186,250 | 27.3 | 240 | AY380826 |
|  | LCDV-1 | 102,653 | 29.1 | 195 | L63545 |
| ***Iridovirus*** | IIV-31 | 220,222 | 35.1 | 203 | HF920637 |
|  | IIV-6 | 212,482 | 28.6 | 468 | AF303741 |
|  | IIV-30 | 198,533 | 30.9 | 177 | HF920636 |
|  | IIV-22 | 197,693 | 28.1 | 167 | HF920633 |
|  | IIV-9 | 205,791 | 30.9 | 191 | GQ918152 |
|  | IIV-25 | 204,815 | 30.3 | 177 | HF920635 |
|  | AMIV | 163,023 | 39.0 | 148 | KF938901 |
| ***Chloriridovirus*** | IIV-3 | 191,100 | 47.9 | 126 | DQ643392 |

**Supplemental file 2.** The primers using to amplify the SHIV genome. (Primers listed in this table were not filtered for specificity and sensitivity.)

| Primers | | 5' position | Primer sequence | Primer length | Product (bp) |
| --- | --- | --- | --- | --- | --- |
| F1 | 165183 | | CAAATCCCACATGCCCGAGTA | 21 | 2174 |
| R1 | 1547 | | ATTGGACGAGTGTGTGTTTGA | 21 |  |
| F2 | 1375 | | CACGAGTTGTCGTTTACGATTC | 22 | 525 |
| R2 | 1900 | | CACGATTTGGCAATTCAAACAA | 22 |  |
| F3 | 1681 | | TTCGTTTTTCATTTTCACATAAAC | 24 | 3195 |
| R3 | 4876 | | TTCGTTCAGTATTGCAGATTC | 21 |  |
| F4 | 4781 | | TTTCCTTTTCAATATTATCGG | 21 | 3104 |
| R4 | 7885 | | ACATGTTTGTAACTGGAAAAA | 21 |  |
| F5 | 7614 | | AATGACAAATTGTAAAATTCCTGT | 24 | 487 |
| R5 | 8101 | | TGATGGAATTTCCAATGTATGGAG | 24 |  |
| F6 | 7898 | | AAACATGTCTTTGACGGCAC | 20 | 490 |
| R6 | 8388 | | GAAGCCCAAGCCGAGAAATA | 20 |  |
| F7 | 8154 | | TTGGCCTTTTTTTCCGTGATG | 21 | 3805 |
| R7 | 11959 | | GCAAGCAGCCGAAAATGAAAT | 21 |  |
| F8 | 11868 | | AATCCTGAAAAGGTAGGACTT | 21 | 3809 |
| R8 | 15677 | | CAAGAACAAGGTTGTAGTCGG | 21 |  |
| F9 | 15365 | | AGAAGTCATCTCTGTTACGA | 20 | 610 |
| R9 | 15975 | | AAACAAGGCCCAAATCATAG | 20 |  |
| F10 | 15802 | | GACTGGTGCAACATCCTTCTT | 21 | 2621 |
| R10 | 18423 | | TTAAGTCTACGATTAAGTCTACGA | 24 |  |
| F11 | 17950 | | AGGGTGTCTCCAGTGGTTTC | 20 | 615 |
| R11 | 18565 | | TTTTCAAATGGTCTCGAATG | 20 |  |
| F12 | 18447 | | TCGTAGACTTAATCCCAGTTG | 21 | 3447 |
| R12 | 21894 | | TCTTCAATGAACGTGTTTTCC | 21 |  |
| F13 | 21377 | | ATGGAAAATAACGAACATGG | 20 | 769 |
| R13 | 22146 | | ACCCCATTCATGTCAATCAT | 20 |  |
| F14 | 22010 | | TTTGTTTTTCTCCTATAGA | 19 | 1786 |
| R14 | 23796 | | CATTGCCAAAATTTATCCAAC | 21 |  |
| F15 | 23650 | | AATATGGCGGAATCAGTAAACA | 22 | 748 |
| R15 | 24398 | | GATATCCCCTTCATTGTCTGGA | 22 |  |
| F16 | 24069 | | ATGGTAATGTGTCTGCGAAC | 20 | 1888 |
| R16 | 25957 | | CGTTTCATATCCAAACGGTG | 20 |  |
| F17 | 25745 | | GCGTGTCATGTGTCCACCGT | 20 | 1125 |
| R17 | 26870 | | TGGTTGGTTTGGATGTAGTA | 20 |  |
| F18 | 26630 | | GCACACACCGGGATATACAT | 20 | 1066 |
| R18 | 27696 | | GTGATTGGTGTTTCAGTGGG | 20 |  |
| F19 | 27474 | | TCAGACCCTTCACTTGAAAT | 20 | 755 |
| R19 | 28229 | | CATCTGGAATCAATTGGTTT | 20 |  |
| F20 | 27577 | | AACCCACAGAACCTTCAATTC | 21 | 1789 |
| R20 | 29366 | | TCCCCCAAATCAAAGATAGGA | 21 |  |
| F21 | 29154 | | AGGGAAATGTATTCGAACGT | 20 | 519 |
| R21 | 29673 | | TAGATTTCAGACGAGGAGAA | 20 |  |
| F22 | 29424 | | AGGAATGTTATCACATTTGAA | 21 | 4814 |
| R22 | 34238 | | CAGAATTGAAAAAGAGAGGAT | 21 |  |
| F23 | 34024 | | CCATTTAGTTTTCCAACATT | 20 | 513 |
| R23 | 34537 | | CTAAACGGCCATCAACGTAT | 20 |  |
| F24 | 34255 | | TCTGACAAAATTGTACTCTTTCCT | 24 | 1879 |
| R24 | 36134 | | TTCAACAGTTTTTCTTCAATCGTT | 24 |  |
| F25 | 35866 | | GAACTCATAGTGATATCCCC | 20 | 1386 |
| R25 | 37252 | | AATTTATCCAACATGAACGT | 20 |  |
| F26 | 37223 | | CGAGATTGATACGTTCATGT | 20 | 510 |
| R26 | 37733 | | CATCTTCTTTACGTTCCATT | 20 |  |
| F27 | 37593 | | ATGTATTGCGAGTATACGATT | 21 | 2487 |
| R27 | 40080 | | TCTCGCAATTTTGAAATGCAG | 21 |  |
| F28 | 39974 | | TCAGACGCCTGTAATTTCATTACC | 24 | 2737 |
| R28 | 42711 | | GGTTGAGGAGTGGGTTTGGGT | 21 |  |
| F29 | 42574 | | CCAAATCGTTCAAGATATTGGG | 22 | 683 |
| R29 | 43257 | | TTGAATCAGTAGAGAACACACC | 22 |  |
| F30 | 43079 | | TACACGACGACGAATACGAC | 20 | 1430 |
| R30 | 44509 | | CAAACAACCAACTCATGTAG | 20 |  |
| F31 | 44319 | | TTTGCATTTGATTCAGAAAAA | 21 | 1917 |
| R31 | 46236 | | TATCTGTGTTAAGAGGAGGAA | 21 |  |
| F32 | 45971 | | CCCCAATCCCATCATTAAAG | 20 | 3023 |
| R32 | 48994 | | GGGTTTCCTTGAACTTGACT | 20 |  |
| F33 | 48799 | | CCCGAGGGGTTAGGAAAAAA | 20 | 6302 |
| R33 | 55101 | | GTTTCTGGAATCTCGACACTCA | 22 |  |
| F34 | 54878 | | AGAGGGTGATGAAGTCATACA | 21 | 2375 |
| R34 | 57253 | | TGTGAATCCAAAAAATGTGAA | 21 |  |
| F35 | 57229 | | TGGATTCACATTTTTTGGATT | 21 | 4699 |
| R35 | 61928 | | AATAGAATGTCGTACTCGACC | 21 |  |
| F36 | 61712 | | GAGAAGAACAACTTCTCAAGTCCA | 24 | 600 |
| R36 | 62312 | | TCTGAAGTGCAATGTTCAATTCCC | 24 |  |
| F37 | 61753 | | GCCAAAGTAAAAGCGTATGGG | 21 | 4780 |
| R37 | 66533 | | CATGATGATCCACTGCTTGCT | 21 |  |
| F38 | 65546 | | CAACACTGACAACGATCTTA | 20 | 1352 |
| R38 | 66898 | | CCAATTTCTCATTCGCGTCA | 20 |  |
| F39 | 66653 | | TATTGCGAATACACGATTCAT | 21 | 3304 |
| R39 | 69957 | | CGTGATTGTTTTGTAGGTTGT | 21 |  |
| F40 | 69533 | | TGTTTTCAGAGATGTTTGGGAATT | 24 | 1255 |
| R40 | 70788 | | TAGACGGGTGGATCCATGACTGAG | 24 |  |
| F41 | 70532 | | ACAAGAGCTTTTAGATTGGGC | 21 | 5072 |
| R41 | 75604 | | TTTGTGAAAACGTTGACTGGA | 21 |  |
| F42 | 75354 | | TTTTCCAAAATTTCATTACCCTTC | 24 | 592 |
| R42 | 75946 | | TCAACAATTTTTTCAAATCTGGGC | 24 |  |
| F43 | 75725 | | TTCCAAATCAAAAACAAGACC | 21 | 2822 |
| R43 | 78547 | | CAAATGCTGTCGTAAATGAAT | 21 |  |
| F44 | 78283 | | ACATCGAAGGTCTCTTGTTG | 20 | 550 |
| R44 | 78833 | | CCTCTCCTCCGAATTGGGCA | 20 |  |
| F45 | 78600 | | CTCGAAAACTATTTACCCAAT | 21 | 1829 |
| R45 | 80429 | | GGGAAATATGAGAGTGAAAATAC | 23 |  |
| F46 | 80249 | | CTCAGAGAATCTTATACGAGTT | 22 | 3540 |
| R46 | 83789 | | GAGGATTCCTATACGGATGAAT | 22 |  |
| F47 | 83661 | | TAAAATTTCGTATTCGTTATCGGT | 24 | 672 |
| R47 | 84333 | | CGAATCTGAACTCGTCAAGCTCGT | 24 |  |
| F48 | 84148 | | AAGAGAAATCGGACGAATGGG | 21 | 2245 |
| R48 | 86393 | | AACGTTTAGCGTCTGCAACGA | 21 |  |
| F49 | 86024 | | TTTGATAACACTGAGAAGATGCGT | 24 | 684 |
| R49 | 86708 | | AAAGTTCTTCTTTTGTTTTGGCTT | 24 |  |
| F50 | 86565 | | ATCTCGCACACCATCAAGACAA | 22 | 417 |
| R50 | 86982 | | CTTTGGGGGTTTGGGTGATGAA | 22 |  |
| F51 | 86838 | | AAAACCAAATTGCGTGCAAA | 20 | 2414 |
| R51 | 89252 | | ATTTATCCAACATGAACGTATCAA | 24 |  |
| F52 | 89086 | | GCCCAATATGGCAGAACAAG | 20 | 2433 |
| R52 | 91519 | | CGATTCGCTTTCGGATACCC | 20 |  |
| F53 | 91409 | | CTGAAACACCTCAAGGAAAA | 20 | 3482 |
| R53 | 94891 | | TGCAGTTCTCGAAAAAGAGC | 20 |  |
| F54 | 94547 | | GAGCGAAGAAAAATTTAAATACAT | 24 | 3790 |
| R54 | 98337 | | TCTATCCAAAACAACGTGCTTTCC | 24 |  |
| F55 | 98137 | | CAAAACTGTTGGTATCGAGT | 20 | 455 |
| R55 | 98592 | | GAATCGTCCGCGTCTATTTT | 20 |  |
| F56 | 98432 | | TGTGGGGTTACCCGAACCAG | 20 | 2032 |
| R56 | 100464 | | CCATTCTGGCCTCTTTTTGG | 20 |  |
| F57 | 100324 | | GAGATTGCCAGAGATCGAGA | 20 | 4106 |
| R57 | 104430 | | TGGATATACGACATCAAAGGACTA | 24 |  |
| F58 | 104261 | | GCGAATACACACTAACGCAAA | 21 | 2681 |
| R58 | 106942 | | TGAAGGTGGTGAACTGATTCC | 21 |  |
| F59 | 106836 | | TATTTCTTCAGCAATGCGTAT | 21 | 2804 |
| R59 | 109640 | | TCTTATTTCCCGAATCATAGAT | 22 |  |
| F60 | 109442 | | CGAAGGAACAAAGGTTTTGG | 20 | 1386 |
| R60 | 110828 | | GCCTGGATGTATTTTCGAGA | 20 |  |
| F61 | 110662 | | AGAAATTGATGAAATCTGCAGAAG | 24 | 676 |
| R61 | 111338 | | TTGTAATCTCCCATTCTGTTATTT | 24 |  |
| F62 | 111122 | | TGGGAAAAAGGGAGTTGTGTT | 21 | 2288 |
| R62 | 113410 | | GAATATTTTTTCACTCAAACGATT | 24 |  |
| F63 | 113271 | | CGTATGTATCATCATCGAAAAT | 22 | 1594 |
| R63 | 114865 | | TTTCCACTTTCAATGGAATCGG | 22 |  |
| F64 | 114712 | | ACATATAAAGGATGGTTCAACAAA | 24 | 3927 |
| R64 | 118639 | | CCGTAGAAACTACTGTGGTGTATG | 24 |  |
| F65 | 118490 | | TATATGATCTGGTTGCACCG | 20 | 5058 |
| R65 | 123548 | | GAATTTCTTCATCTCATACATCTG | 24 |  |
| F66 | 123261 | | TGCCGAATCGGATGAATCCGT | 21 | 2765 |
| R66 | 126026 | | AATTTATCCAACATGAACGTATCA | 24 |  |
| F67 | 125973 | | CAAATATATTGAAACGGTTAAATT | 24 | 4462 |
| R67 | 130435 | | AACTGATGATAAAATTCGTTTGGG | 24 |  |
| F68 | 129994 | | TCATTTCTCATCGTTGTCCAC | 21 | 1779 |
| R68 | 131773 | | GTTCTATTTCACGATCTTGTT | 21 |  |
| F69 | 131526 | | GAAAAGGAGCGAATGGAAAA | 20 | 3085 |
| R69 | 134611 | | CATCGCTGCAATGGGTATCA | 20 |  |
| F70 | 134299 | | CTTTTGGAATATCTTCTTTTGGA | 23 | 3776 |
| R70 | 138075 | | TTTGCGACACAGCTGCTTTAA | 21 |  |
| F71 | 137879 | | AAAAGCCCATAATGGCAGAA | 20 | 1214 |
| R71 | 139093 | | ATTCCACATGCTCCTGTGAA | 20 |  |
| F72 | 138844 | | CACAAGATTGGTTTGGTTAAAATG | 24 | 1399 |
| R72 | 140243 | | GAAGATGAACCCGTCAAGGAA | 21 |  |
| F73 | 140136 | | TCGTCTTGGGAGGGCTCGTCT | 21 | 4399 |
| R73 | 144535 | | TTGATGCTACTGATGGTGGTC | 21 |  |
| F74 | 144278 | | GCCATGTTCCCCTGTGGAAA | 20 | 897 |
| R74 | 145175 | | CTGCTATTAAAGCGGCAAAG | 20 |  |
| F75 | 145151 | | ACCAGTCTTGGCTTCACCTTCACCC | 25 | 807 |
| R75 | 145958 | | TGATGATCTTCTGATCCCAAAATCC | 25 |  |
| F76 | 145735 | | CGATATCACTTCCAACCTGAG | 21 | 2915 |
| R76 | 148650 | | TGAAGCCTCTTTCCTTCTTCG | 21 |  |
| F77 | 148475 | | AGAAGGAAAAGAAGGAAAACACGC | 24 | 4958 |
| R77 | 153433 | | TTTTAAAGGAAAATAAATCCGAAC | 24 |  |
| F78 | 152675 | | CGTTGCATGATTTACTCGTTTT | 22 | 9101 |
| R78 | 161776 | | GAACAAGGGGTCTTGTAAATAG | 22 |  |
| F79 | 161578 | | GGCCCTGGATTATGTACAGT | 20 | 3814 |
| R79 | 165392 | | GCGACTACAAGCAGTAGTAA | 20 |  |

**Supplemental file 3.** ORF predictionsa in the IIV31 genome. R express the ORFs in forward orientation and L express the ORFs in reverse orientation.

| ORF | Length  (aa) | Initial  position | Terminal  position | Predicted  Structure  or function | Best match | | | |
| --- | --- | --- | --- | --- | --- | --- | --- | --- |
|  |  |  |  |  | Species | Accession number | Identity  (%) | score |
| 1R | 182 | 1 | 549 | hypothetical protein | Invertebrate iridescent virus 6 | AAB94431.1 | 26.21 | 27.7 |
| 2L | 368 | 586 | 1692 | DNA binding packing | Lymphocystis disease virus - isolate China | AAU11024.1 | 48.24 | 152 |
| 3L | 137 | 1745 | 2158 | hypothetical protein | Invertebrate iridescent virus 6 | AAK82275.1 | 50 | 23.5 |
| 4L | 167 | 2155 | 2658 | hypothetical protein | Invertebrate iridovirus 25 | CCV02128.1 | 56.52 | 34.3 |
| 5L | 69 | 2686 | 2895 | hypothetical protein | Singapore grouper iridovirus | AAS18171.1 | 28.57 | 23.5 |
| 6L | 445 | 2940 | 4277 | DNA topoisomerase mitochondrial | Lymphocystis disease virus Sa | AOC55150.1 | 50 | 25.8 |
| 7R | 111 | 4271 | 4606 | hypothetical protein | Invertebrate iridescent virus 30 | CCV02339.1 | 42.86 | 24.6 |
| 8L | 131 | 4770 | 5165 | hypothetical protein | Invertebrate iridescent virus 6 | AAB94478.1 | 36.84 | 24.3 |
| 9L | 105 | 5202 | 5519 | hypothetical protein | Grouper iridovirus | AAV91057.1 | 42.22 | 28.5 |
| 10L | 158 | 5549 | 6025 | hypothetical protein | Lymphocystis disease virus - isolate China | AAU10919.1 | 50 | 34.7 |
| 11L | 1167 | 6062 | 9565 | hypothetical protein | Armadillidium vulgare iridescent virus | CCV02483.1 | 54.05 | 40 |
| 12R | 466 | 9650 | 11050 | Phosphotransferase | Lymphocystis disease virus - isolate China | AAU10904.1 | 42.86 | 26.6 |
| 13R | 287 | 11052 | 11915 | hypothetical protein | Wiseana iridescent virus | ADO00445.1 | 24.24 | 24.3 |
| 14L | 165 | 11955 | 12452 | inactivated thioredoxin glutaredoxin | Wiseana iridescent virus | ADO00498.1 | 41.18 | 26.2 |
| 15R | 200 | 12523 | 13125 | hypothetical protein | Armadillidium vulgare iridescent virus | CCV02517.1 | 33.33 | 23.5 |
| 16R | 218 | 13190 | 13846 | SWIB MDM2 domain-containing | Armadillidium vulgare iridescent virus | CCV02475.1 | 44.83 | 27.7 |
| 17L | 476 | 13943 | 15373 | serine threonine- kinase VRK1-like | Anopheles minimus irodovirus | AHL67528.1 | 61.9 | 25.8 |
| 18L | 326 | 15529 | 16509 | hypothetical protein | Frog virus 3 | AAT09708.1 | 41.94 | 30 |
| 19R | 551 | 16717 | 18372 | zinc finger CCCH domain-containing 37 | Invertebrate iridovirus 25 | CCV02030.1 | 50 | 26.9 |
| 20L | 642 | 18562 | 20490 | hypothetical protein | Invertebrate iridovirus 22 | CCV01749.1 | 32.86 | 27.7 |
| 21R | 218 | 20609 | 21265 | hypothetical protein | Ranavirus maximus | ANZ57182.1 | 47.37 | 24.3 |
| 22R | 172 | 21393 | 21911 | hypothetical protein | Invertebrate iridovirus 22 | CCV01835.1 | 52 | 25 |
| 23R | 495 | 22053 | 23540 | XRN1 5 -3 exoribonuclease | Invertebrate iridescent virus 6 | AAK81943.1 | 40.43 | 28.1 |
| 24R | 123 | 23653 | 24024 | hypothetical protein | Armadillidium vulgare iridescent virus | CCV02541.1 | 55.56 | 38.5 |
| 25R | 249 | 24021 | 24770 | hypothetical protein | Wiseana iridescent virus | ADO00396.1 | 60 | 25.4 |
| 26L | 115 | 24803 | 25150 | Thioredoxin-2 | Armadillidium vulgare iridescent virus | CCV02415.1 | 29.51 | 33.1 |
| 27L | 95 | 25340 | 25627 | hypothetical protein | Tiger frog virus | ABB92307.1 | 29.51 | 23.1 |
| 28L | 168 | 25634 | 26140 | hypothetical protein | Lymphocystis disease virus Sa | AOC55213.1 | 43.24 | 25.4 |
| 29R | 541 | 26214 | 27839 | Ca2+-binding RTX toxin-related | Grouper iridovirus | AAV91041.1 | 51.11 | 26.6 |
| 30R | 443 | 27933 | 29264 | group I intron endonuclease | Invertebrate iridovirus 22 | CCV01681.1 | 45 | 33.5 |
| 31L | 363 | 29307 | 30398 | DNA repair RAD2 | Armadillidium vulgare iridescent virus | CCV02535.1 | 43.55 | 90.1 |
| 32L | 134 | 30450 | 30854 | hypothetical protein | Aedes taeniorhynchus iridescent virus | ABF82143.1 | 38.24 | 25 |
| 33R | 137 | 31008 | 31421 | hypothetical protein | Lymphocystis disease virus Sa | AOC55091.1 | 29.17 | 25.4 |
| 34L | 201 | 31470 | 32075 | hypothetical protein | Anopheles minimus irodovirus | AHL67590.1 | 57.14 | 27.3 |
| 35R | 433 | 32074 | 33375 | hypothetical protein | Invertebrate iridovirus 22 | CCV01742.1 | 51.22 | 32 |
| 36L | 200 | 33714 | 34316 | Deoxynucleoside kinase | Invertebrate iridovirus 22 | CCV01819.1 | 53.33 | 24.3 |
| 37L | 81 | 34396 | 34641 | hypothetical protein | Singapore grouper iridovirus | AAS18040.1 | 25.45 | 23.1 |
| 38R | 659 | 35073 | 37052 | DNA double-strand break repair rad50 ATPase-like | Lymphocystis disease virus Sa | AOC55245.1 | 44.44 | 28.1 |
| 39R | 402 | 37107 | 38315 | hypothetical protein | Wiseana iridescent virus | ADO00493.1 | 50 | 33.9 |
| 40R | 261 | 38414 | 39199 | proliferating cell nuclear antigen | Wiseana iridescent virus | ADO00452.1 | 44.83 | 24.6 |
| 41R | 112 | 39234 | 39572 | hypothetical protein | Lymphocystis disease virus Sa | AOC55213.1 | 62.5 | 26.6 |
| 42R | 140 | 39565 | 39987 | hypothetical protein |  |  |  |  |
| 43L | 194 | 40019 | 40603 | Serpentine type 7TM GPCR chemoreceptor Srd |  |  |  |  |
| 44L | 350 | 40784 | 41836 | DNA repair exonuclease D subunit | Invertebrate iridovirus 22 | CCV01805.1 | 55 | 25.8 |
| 45L | 129 | 41863 | 42252 | Ribonuclease III | Invertebrate iridovirus 22 | CCV01792.1 | 53.57 | 24.3 |
| 46R | 331 | 42304 | 43299 | hypothetical protein | Invertebrate iridovirus 22 | CCV01815.1 | 50 | 83.6 |
| 47R | 266 | 43335 | 44135 | ribonuclease III | Lymphocystis disease virus - isolate China | AAU11030.1 | 41.41 | 157 |
| 48L | 76 | 44342 | 44572 | hypothetical protein |  |  |  |  |
| 49L | 152 | 44969 | 45427 | hypothetical protein | Invertebrate iridescent virus 30 | CCV02353.1 | 50 | 32.7 |
| 50L | 162 | 45440 | 45928 | hypothetical protein | Infectious spleen and kidney necrosis virus | AAL98766.1 | 47.62 | 25.4 |
| 51R | 525 | 46085 | 47662 | Papain ase | Lymphocystis disease virus - isolate China | AAU11066.1 | 41.13 | 273 |
| 52R | 285 | 47699 | 48556 | hypothetical protein | Invertebrate iridovirus 25 | CCV02123.1 | 37.84 | 25.4 |
| 53R | 57 | 48610 | 48783 | hypothetical protein |  |  |  |  |
| 54R | 121 | 49142 | 49507 | hypothetical protein | Wiseana iridescent virus | ADO00493.1 | 50 | 37.4 |
| 55R | 326 | 49532 | 50512 | NAD-dependent DNA ligase | Wiseana iridescent virus | ADO00488.1 | 50 | 28.1 |
| 56R | 783 | 50604 | 52955 | DNA-directed RNA polymerase II largest subunit | Lymphocystis disease virus - isolate China | AAS47822.1 | 40.31 | 479 |
| 57R | 546 | 52948 | 54588 | DNA-dependent RNA polymerase II largest subunit | Invertebrate iridescent virus 6 | AAB94477.1 | 48.25 | 113 |
| 58R | 124 | 54624 | 54998 | hypothetical protein | Invertebrate iridescent virus 6 | AAB94472.1 | 28.85 | 26.9 |
| 59L | 175 | 55453 | 55980 | hypothetical protein | Lymphocystis disease virus - isolate China | AAU10860.1 | 36.72 | 63.9 |
| 60R | 377 | 56055 | 57188 | hypothetical protein | Armadillidium vulgare iridescent virus | CCV02505.1 | 56 | 25.8 |
| 61R | 293 | 57335 | 58216 | hypothetical protein | Invertebrate iridovirus 25 | CCV02174.1 | 38.24 | 30.8 |
| 62L | 304 | 58889 | 59803 | Phytanoyl- dioxygenase | Ambystoma tigrinum virus | YP_003835.1 | 34.69 | 24.6 |
| 63R | 423 | 59903 | 61174 | hypothetical protein | Armadillidium vulgare iridescent virus | CCV02431.1 | 38.24 | 26.9 |
| 64R | 543 | 61216 | 62847 | NTPase helicase | Tortoise ranavirus | AJR29279.1 | 52.17 | 27.7 |
| 65R | 606 | 62994 | 64814 | NAD-dependent DNA ligase | Invertebrate iridovirus 25 | CCV02188.1 | 35.98 | 288 |
| 66R | 182 | 64873 | 65421 | hypothetical protein | Lymphocystis disease virus Sa | AOC55185.1 | 48.39 | 24.6 |
| 67R | 160 | 65526 | 66008 | hypothetical protein | Lymphocystis disease virus - isolate China | AAU10886.1 | 33.33 | 24.3 |
| 68R | 479 | 66152 | 67591 | hypothetical protein | Tiger frog virus | ABB92270.1 | 55.56 | 26.9 |
| 69R | 1142 | 67578 | 71006 | DNA-directed RNA polymerase II second largest subunit | Tortoise ranavirus | AJR29268.1 | 41.06 | 653 |
| 70R | 461 | 71077 | 72462 | ATP-dependent helicase | Invertebrate iridescent virus 30 | CCV02257.1 | 35.71 | 26.2 |
| 71L | 210 | 72671 | 73303 | hypothetical protein | Armadillidium vulgare iridescent virus | CCV02483.1 | 29.87 | 28.1 |
| 72R | 160 | 73464 | 73946 | NUDIX family | Testudo hermanni ranavirus | AJR29142.1 | 40 | 25 |
| 73R | 406 | 74068 | 75288 | immediate early ICP-46 | Lymphocystis disease virus - isolate China | AAU11006.1 | 28.15 | 79.7 |
| 74L | 77 | 75400 | 75633 | ubiquitin | Singapore grouper iridovirus | AAS18117.1 | 77.92 | 125 |
| 75R | 155 | 75669 | 76136 | hypothetical protein | Frog virus 3 | AAT09708.1 | 46.15 | 27.7 |
| 76R | 162 | 76162 | 76650 | hypothetical protein | Aedes taeniorhynchus iridescent virus | ABF82046.1 | 34.41 | 26.6 |
| 77R | 1080 | 76740 | 79982 | DNA topoisomerase II | Anopheles minimus irodovirus | AHL67621.1 | 40.81 | 516 |
| 78R | 149 | 80012 | 80461 | hypothetical protein |  |  |  |  |
| 79L | 461 | 80669 | 82054 | hypothetical protein | Wiseana iridescent virus | ADO00382.1 | 36.36 | 28.1 |
| 80L | 182 | 82101 | 82649 | hypothetical protein | Lymphocystis disease virus Sa | AOC55132.1 | 33.33 | 24.3 |
| 81L | 154 | 82687 | 83151 | hypothetical protein | Armadillidium vulgare iridescent virus | CCV02457.1 | 31.25 | 25 |
| 82L | 236 | 83157 | 83867 | hypothetical protein | Lymphocystis disease virus - isolate China | AAU11001.1 | 48.15 | 25.8 |
| 83R | 21 | 83970 | 84035 | hypothetical protein |  |  |  |  |
| 84L | 92 | 84456 | 84734 | hypothetical protein | Lymphocystis disease virus - isolate China | AAU10958.1 | 47.62 | 21.2 |
| 85L | 220 | 84731 | 85393 | hypothetical protein | Armadillidium vulgare iridescent virus | CCV02373.1 | 22.58 | 26.6 |
| 86R | 173 | 85530 | 86051 | DNA-directed RNA polymerases II and IV subunit 5A | Lymphocystis disease virus Sa | AOC55189.1 | 43.75 | 21.9 |
| 87R | 784 | 86125 | 88479 | neurofilament triplet H1 | Invertebrate iridescent virus 30 | CCV02206.1 | 46 | 26.9 |
| 88R | 125 | 88530 | 88907 | transcription elongation factor sii | Invertebrate iridescent virus 30 | CCV02278.1 | 42.17 | 69.3 |
| 89R | 397 | 89093 | 90286 | hypothetical protein | Wiseana iridescent virus | ADO00493.1 | 43.75 | 30 |
| 90R | 1021 | 90379 | 93444 | tyrosine kinase | Invertebrate iridescent virus 6 | AAB94478.1 | 40.65 | 366 |
| 91R | 150 | 93481 | 93933 | hypothetical protein | Invertebrate iridovirus 22 | CCV01837.1 | 28 | 24.3 |
| 92R | 184 | 93954 | 94508 | hypothetical protein | Anopheles minimus irodovirus | AHL67535.1 | 46.15 | 23.1 |
| 93R | 170 | 94545 | 95057 | hypothetical protein | Invertebrate iridovirus 22 | CCV01719.1 | 34.88 | 25.8 |
| 94L | 508 | 95054 | 96580 | serine threonine kinase | Invertebrate iridovirus 22 | CCV01760.1 | 59.09 | 32 |
| 95L | 471 | 96609 | 98024 | hypothetical protein | Armadillidium vulgare iridescent virus | CCV02436.1 | 43.48 | 28.1 |
| 96R | 192 | 98088 | 98666 | Thymidylate kinase | Invertebrate iridescent virus 6 | AAK82112.1 | 40 | 164 |
| 97R | 144 | 98683 | 99117 | hypothetical protein | Lymphocystis disease virus - isolate China | AAU10893.1 | 41.38 | 23.5 |
| 98R | 188 | 99149 | 99715 | N1R p28 | Invertebrate iridescent virus 6 | AAK82105.1 | 42.5 | 27.7 |
| 99R | 421 | 99781 | 101046 | hypothetical protein | Armadillidium vulgare iridescent virus | CCV02541.1 | 52 | 30.4 |
| 100R | 235 | 101268 | 101975 | BRO-like gene | Invertebrate iridovirus 22 | CCV01681.1 | 45.83 | 45.4 |
| 101R | 65 | 102178 | 102375 | hypothetical protein | Invertebrate iridescent virus 6 | AAK82328.1 | 52 | 22.7 |
| 102R | 384 | 102384 | 103538 | hypothetical protein | Invertebrate iridovirus 22 | CCV01681.1 | 50 | 47.4 |
| 103L | 289 | 103596 | 104465 | hypothetical protein | Invertebrate iridescent virus 6 | AAK82075.1 | 40 | 41.2 |
| 104R | 179 | 104490 | 105029 | Uvr REP helicase | Lymphocystis disease virus Sa | AOC55174.1 | 62.96 | 146 |
| 105R | 294 | 105052 | 105936 | patatin-like phospholipase | Invertebrate iridescent virus 6 | AAK82227.1 | 40 | 28.1 |
| 106R | 101 | 106213 | 106518 | hypothetical protein | Grouper iridovirus | AAV91028.1 | 45.95 | 27.3 |
| 107L | 212 | 106572 | 107210 | hypothetical protein | Aedes taeniorhynchus iridescent virus | ABF82049.1 | 37.5 | 24.3 |
| 108L | 211 | 107355 | 107990 | hypothetical protein | Wiseana iridescent virus | ADO00461.1 | 24.62 | 24.3 |
| 109L | 161 | 108042 | 108527 | hypothetical protein | Armadillidium vulgare iridescent virus | CCV02376.1 | 30 | 23.5 |
| 110R | 1049 | 108648 | 111797 | DNA polymerase | Invertebrate iridovirus 22 | CCV01678.1 | 44.3 | 305 |
| 111R | 108 | 111850 | 112176 | hypothetical protein | Aedes taeniorhynchus iridescent virus | ABF82035.1 | 38.71 | 22.7 |
| 112R | 285 | 112220 | 113077 | hypothetical protein | Wiseana iridescent virus | ADO00493.1 | 46.67 | 34.3 |
| 113L | 184 | 113707 | 114261 | Haloacid dehalogenase-like hydrolases | Armadillidium vulgare iridescent virus | CCV02410.1 | 49.7 | 145 |
| 114R | 903 | 114762 | 117473 | D5 family NTPase ATPase | Epizootic haematopoietic necrosis virus | ACO25275.1 | 51.44 | 301 |
| 115L | 185 | 117533 | 118090 | hypothetical protein | Soft-shelled turtle iridovirus | ACF42253.1 | 40.74 | 24.6 |
| 116L | 188 | 118116 | 118682 | hypothetical protein | Invertebrate iridescent virus 6 | AAK81999.1 | 58.33 | 22.7 |
| 117R | 241 | 118744 | 119469 | hypothetical protein |  |  |  |  |
| 118R | 305 | 119503 | 120420 | hypothetical protein | European catfish virus | AMZ04912.1 | 26.32 | 35.4 |
| 119L | 256 | 120485 | 121255 | ATpase 3 | Lymphocystis disease virus - isolate China | AAU10959.1 | 51.24 | 258 |
| 120R | 107 | 121382 | 121705 | hypothetical protein | Short-finned eel ranavirus | ANK58076.1 | 50 | 23.5 |
| 121R | 112 | 122338 | 122676 | hypothetical protein | Wiseana iridescent virus | ADO00532.1 | 58.82 | 23.1 |
| 122L | 124 | 122719 | 123093 | thiol reductase thioredoxin | Singapore grouper iridovirus | AAS18116.1 | 45 | 25.8 |
| 123L | 225 | 123117 | 123794 | hypothetical protein | Aedes taeniorhynchus iridescent virus | ABF82037.1 | 45.45 | 25.4 |
| 124R | 643 | 123854 | 125785 | cell surface | Lymphocystis disease virus - isolate China | AAU11010.1 | 33.33 | 30 |
| 125R | 453 | 125878 | 127239 | hypothetical protein | Lymphocystis disease virus Sa | AOC55209.1 | 55 | 28.9 |
| 126L | 64 | 127278 | 127472 | hypothetical protein | Armadillidium vulgare iridescent virus | CCV02497.1 | 50 | 22.7 |
| 127R | 116 | 127516 | 127866 | hypothetical protein | Invertebrate iridescent virus 6 | AAK82208.1 | 26.32 | 26.2 |
| 128R | 192 | 128008 | 128586 | hypothetical protein | Turbot reddish body iridovirus | ADE34435.1 | 36.6 | 103 |
| 129L | 526 | 128637 | 130217 | hypothetical protein | Armadillidium vulgare iridescent virus | CCV02504.1 | 32.61 | 27.7 |
| 130R | 222 | 130267 | 130935 | membrane-associated phosphatidylinositol transfer 1 | Aedes taeniorhynchus iridescent virus | ABF82050.1 | 70 | 24.3 |
| 131R | 199 | 130974 | 131573 | hypothetical protein | Lymphocystis disease virus Sa | AOC55236.1 | 40 | 24.6 |
| 132R | 51 | 131739 | 131894 | nucleolar preribosomal assembly |  |  |  |  |
| 133L | 228 | 132008 | 132694 | hypothetical protein | Infectious spleen and kidney necrosis virus | AAL98786.1 | 33.33 | 24.6 |
| 134L | 191 | 132894 | 133469 | hypothetical protein | Armadillidium vulgare iridescent virus | CCV02554.1 | 50 | 24.3 |
| 135L | 183 | 133627 | 134178 | hypothetical protein | Invertebrate iridovirus 25 | CCV02142.1 | 29.27 | 24.6 |
| 136L | 129 | 134231 | 134620 | N-acetylmuramoyl-L-alanine amidase | Short-finned eel ranavirus | ANK58083.1 | 39.47 | 29.6 |
| 137R | 311 | 134706 | 135641 | RING finger 148-like | Lymphocystis disease virus Sa | AOC55194.1 | 48 | 25 |
| 138R | 170 | 136135 | 136647 | deoxyuridine 5 -triphosphate nucleotidohydrolase | Tiger frog virus | AAL77806.1 | 54.84 | 129 |
| 139R | 160 | 136709 | 137191 | hypothetical protein | Short-finned eel ranavirus | ANK58131.1 | 71.43 | 23.9 |
| 140R | 172 | 137307 | 137825 | hypothetical protein | Invertebrate iridovirus 22 | CCV01807.1 | 33.33 | 23.9 |
| 141R | 62 | 137890 | 138078 | hypothetical protein | Armadillidium vulgare iridescent virus | CCV02398.1 | 56.25 | 23.5 |
| 142R | 339 | 138172 | 139191 | hypothetical protein | Invertebrate iridescent virus 30 | CCV02291.1 | 44.71 | 79.3 |
| 143R | 146 | 139264 | 139704 | hypothetical protein |  |  |  |  |
| 144L | 109 | 139746 | 140075 | hypothetical protein | Aedes taeniorhynchus iridescent virus | ABF82154.1 | 52.63 | 26.9 |
| 145L | 213 | 140120 | 140761 | hypothetical protein | Lymphocystis disease virus - isolate China | AAU10990.1 | 45.16 | 27.3 |
| 146R | 1026 | 140864 | 143944 | NTPase | Aedes taeniorhynchus iridescent virus | ABF82044.1 | 35.9 | 28.5 |
| 147R | 237 | 143975 | 144688 | hypothetical protein | Invertebrate iridescent virus 6 | AAB94477.1 | 39.02 | 27.7 |
| 148L | 477 | 145055 | 146488 | Major Capsid Protein | Armadillidium vulgare iridescent virus | CCV02506.1 | 45.94 | 433 |
| 149L | 177 | 146482 | 147015 | hypothetical protein | Singapore grouper iridovirus | AAS18053.1 | 44 | 24.3 |
| 150R | 305 | 147112 | 148029 | rgv late gene | Lymphocystis disease virus - isolate China | AAU10945.1 | 34.1 | 128 |
| 151R | 194 | 148099 | 148683 | high mobility group | Lymphocystis disease virus - isolate China | AAU10919.1 | 38.46 | 28.9 |
| 152L | 352 | 148726 | 149784 | NTPase helicase | Lymphocystis disease virus Sa | AOC55267.1 | 45.71 | 27.3 |
| 153L | 185 | 149974 | 150531 | hypothetical protein | Invertebrate iridescent virus 6 | AAK82245.1 | 45.16 | 28.9 |
| 154L | 187 | 150545 | 151108 | hypothetical protein | Invertebrate iridescent virus 6 | AAK82245.1 | 48.28 | 27.7 |
| 155L | 206 | 151114 | 151734 | hypothetical protein | Invertebrate iridescent virus 6 | AAK82245.1 | 52.63 | 23.9 |
| 156R | 133 | 151821 | 152222 | hypothetical protein | Wiseana iridescent virus | ADO00526.1 | 30.88 | 23.1 |
| 157L | 450 | 152276 | 153628 | helicase | Aedes taeniorhynchus iridescent virus | ABF82138.1 | 46.94 | 103 |
| 158L | 456 | 153653 | 155023 | hypothetical protein | Armadillidium vulgare iridescent virus | CCV02573.1 | 39.53 | 26.9 |
| 159R | 512 | 155084 | 156622 | Lipid membrane | Armadillidium vulgare iridescent virus | CCV02434.1 | 48.28 | 25.4 |
| 160R | 180 | 156653 | 157195 | hypothetical protein | Lymphocystis disease virus Sa | AOC55219.1 | 28 | 22.3 |
| 161R | 223 | 157449 | 158120 | hypothetical protein | Invertebrate iridescent virus 6 | AAB94428.1 | 34.88 | 25 |
| 162R | 181 | 158248 | 158793 | uncharacterized PPE family PPE16-like | Lymphocystis disease virus Sa | AOC55195.1 | 28.89 | 25 |
| 163R | 552 | 158881 | 160539 | hypothetical protein | Lymphocystis disease virus Sa | AOC55132.1 | 25 | 27.3 |
| 164R | 164 | 160628 | 161122 | Appr-1-p processing | Lymphocystis disease virus Sa | AOC55140.1 | 35 | 26.2 |
| 165L | 163 | 161166 | 161657 | hypothetical protein | Lymphocystis disease virus Sa | AOC55132.1 | 50 | 24.3 |
| 166L | 151 | 161715 | 162170 | hypothetical protein | Lymphocystis disease virus Sa | AOC55213.1 | 40.54 | 23.5 |
| 167R | 293 | 162658 | 163539 | hypothetical protein | Wiseana iridescent virus | ADO00491.1 | 30.43 | 25 |
| 168L | 225 | 163593 | 164270 | hypothetical protein | Invertebrate iridovirus 22 | CCV01758.1 | 20.22 | 27.7 |
| 169R | 319 | 164489 | 165448 | myristylated membrane | Invertebrate iridovirus 22 | CCV01765.1 | 40.54 | 197 |
| 170R | 102 | 165426 | 165734 | hypothetical protein | Invertebrate iridovirus 25 | CCV02074.1 | 37.93 | 23.1 |

**Supplemental file 4.** SHIV genes homologous to other iridescent viruses. (*16 of the 27 genes matched 34 iridescent viruses)

| *2L DNA binding packing | *56R DNA-directed RNA polymerase II largest subunit | 104R Hypothetical protein |
| --- | --- | --- |
| *11L Dynein-like beta chain | *57R DNA-dependent RNA polymerase II largest subunit | *110R DNA polymerase |
| *12R Phosphotransferase | *69R DNA-directed RNA polymerase II second largest subunit | *113L Haloacid dehalogenase-like hydrolases |
| 17L Serine threonine- kinase VRK1-like | 70R ATP-dependent helicase | 114R D5 family NTPase ATPase |
| 28L Hypothetical protein | 73R Immediate early ICP-46 | *119L ATpase 3 |
| *31L DNA repair RAD2 | 87R Neurofilament triplet H1 | *146R NTPase |
| 35R Hypothetical protein | 88R Transcription elongation factor sii | *148L Major capsid protein |
| *36L Deoxynucleoside kinase | 90R Tyrosine kinase | 150R rgv late gene |
| *47R Ribonuclease III | *94L Serine threonine kinase | *159R Lipid membrane |
